# Predictors of postoperative delirium in elderly patients following total hip and knee arthroplasty: a systematic review and meta-analysis

**DOI:** 10.1186/s12891-021-04825-1

**Published:** 2021-11-12

**Authors:** Quan Zhou, Xinfeng Zhou, Yijian Zhang, Mingzhuang Hou, Xin Tian, Huilin Yang, Fan He, Xi Chen, Tao Liu

**Affiliations:** 1 Department of Orthopaedics, The First Affiliated Hospital of Soochow University, No. 899 Pinghai Road, Suzhou, 215006 Jiangsu China; 2 Orthopaedic Institute, Medical College, Soochow University, Suzhou, 215007 China; 3 Department of Pathology, The Third Affiliated Hospital of Soochow University, No.185 Juqian Road, Changzhou, 213003 Jiangsu China

**Keywords:** Postoperative delirium, Total joint arthroplasty, Total hip arthroplasty, Total knee arthroplasty, Predictors

## Abstract

**Background:**

Postoperative delirium (POD) is widely reported as a common postoperative complication following total joint arthroplasty (TJA) of the hip and knee in elderly patients, leading to many adverse effects. We sought to investigate predictors of delirium after TJA.

**Methods:**

PubMed, EMBASE, Cochrane Library and Web of Science were searched up to 2020 for studies examining POD following TJA in elderly patients. Pooled odds ratio (OR) and mean difference (MD) of those who experienced delirium compared to those who did not were calculated for each variable. The Newcastle-Ottawa Scale (NOS) was used for the study quality evaluation.

**Results:**

Fifteen studies with 31 potential factors were included. In the primary analysis, 9 factors were associated with POD, comprising advanced age (MD 3.81; 95% confidence interval (CI) 1.80–5.83), dementia (OR 24.85; 95% CI 7.26–85.02), hypertension (OR 2.26; 95% CI 1.31–3.89), diabetes (OR 2.02; 95% CI 1.15–3.55), stroke (OR 14.61; 95% CI 5.26–40.55), psychiatric illness (OR 2.72; 95% CI 1.45–5.08), use of sedative-hypnotics (OR 6.42; 95% CI 2.53–16.27), lower preoperative levels of hemoglobin (MD − 0.56; 95% CI − 0.89−− 0.22), and lower preoperative mini-mental state examination score (MD − 0.40; 95% CI − 0.69−− 0.12). Twelve studies were included in the systematic review, of which 24 factors were additionally correlated with POD using single studies.

**Conclusions:**

Strategies and interventions should be implemented for the elderly patients receiving TJA surgeries with potential predictors identified in this meta-analysis.

**Supplementary Information:**

The online version contains supplementary material available at 10.1186/s12891-021-04825-1.

## Background

Total joint arthroplasty (TJA) remains one of the most effective modern surgical interventions for pain relief and functional recovery. With the aging of the population, the demand for TJA is continually rising, and the accompanying problems are becoming the focus of concerns. It is estimated that the demand for hip arthroplasty procedures is projected to rise by almost twofold in 2030, while that for knee arthroplasty procedures by almost sevenfold [1]. Although the perioperative course is relatively predictable, patients who undergo TJA still suffer from various postoperative complications, especially in elderly patients [2].

Postoperative delirium (POD), as a sudden, transient disturbance of attention, perception, and consciousness, is one of the widely reported complications after TJA [3]. POD is a serious clinical syndrome associated with significant negative consequences, including more hospital-acquired complications, higher mortality rates, progressive cognitive impairments, longer hospital stays, and increased medical costs [4]. The reported incidence of POD in total hip arthroplasty ranges from 8.4 to 18.1%, and 12.8–30.5% in total knee arthroplasty [5]. The disparately reported incidence of delirium after surgery is dependent on diagnostic criteria for delirium, frequency of assessment, and the qualifications and experience of the assessors [5].

Although the pathophysiological mechanism of delirium after TJA remains ambiguous, various predisposing factors which associated with POD have been reported, including preoperative mental status, aging, multiple medical comorbidities, and psychotropic medications [6–11]. However, these potential predictors were limited because most of them were derived from individual studies. In addition, some research results were often inconsistent and even contradictory, which affected the clinical application. So far, there are no definite factors that can effectively predict the occurrence of POD. Candidate predictors to detect whether patients receiving TJA are at imminent risk for POD will be very necessary. They can not only help the construction of risk prediction model and enable early prevention, but also have great significance for clinical prognostication, patients’ stratification, and guiding the further research of this disease. Therefore, we conducted this extensive and comprehensive meta-analysis that examined the available literature to identify the predictors for delirium after TJA.

## Methods

### Search strategy

The search process was based on the Preferred Reporting Items for Systematic reviews and Meta-Analyses (PRISMA) guidelines [12]. From database inception to 25th May 2020, four online databases (EMBASE, PubMed, Cochrane Database, and Web of Science) were exhaustively searched for studies reporting postoperative delirium following TJA in elderly patients. The detailed search strategy used in PubMed is shown in Table S1 (Supplementary Material). Other databases were searched using similar search strategies. In addition, manual reference checking of relevant reviews was performed for possible inclusion. No language restrictions were applied.

### Study selection

**Fig. 1:**
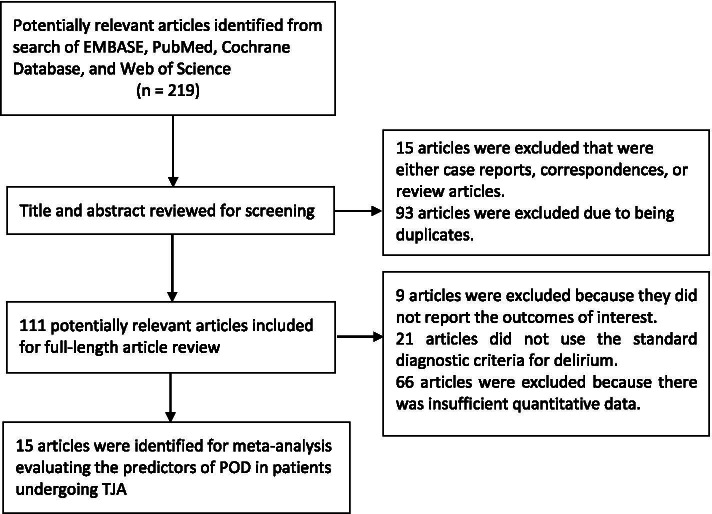
PRISMA flowchart describing the study’s systematic literature search and study selection

The following inclusion criteria were used: (1) The included studies mentioned risk factors associated with postoperative delirium after TJA of the hip or knee (primary or revision); (2) Eligible older patients were 60 years of age or more; (3) Comparison between Delirium and No Delirium groups; (4) Screened for postoperative delirium using either Diagnostic and Statistical Manual of Mental Disorders (DSM) [13] or Confusion Assessment Method (CAM) [14]; (5) Sufficient data could be obtained to estimate the mean difference (MD) or odds ratio (OR) with 95% confidence interval (CI). The specific exclusion criteria are shown in Fig. 1. The retrieval results of this systematic review were independently evaluated by two authors (Q. Z and X.F Z.) by viewing the title/abstract or the full text. If there were any differences between the two authors, the third author (Y.J Z.) would help resolve them until a final consensus was reached.

### Data extraction

All data were independently extracted and recorded by two investigators (Q Z. and X T.) from qualified studies. This process was performed according to a predefined data extraction template. The extracted data was as follows:

Study variables: first author’s name; publication year; study types; country; sample size; numbers of cases and controls; tools of assessing delirium; Newcastle Ottawa Scale (NOS) scores.

Demographic variables: age; sex; education; body mass index (BMI); smoking; alcohol abuse.

Physical status-related variables: preoperative Mini-Mental State Evaluation (MMSE) score; co-morbidities (such as dementia, stroke, hypertension, diabetes, hyperlipidemia, ischemic heart disease, psychiatric illness); surgical complications (such as surgical site infection, deep vein thrombosis).

Surgery-related variables: types of anesthesia; types of operation; operation time; intraoperative blood loss.

Drug-related variables: use of sedative-hypnotics.

Laboratory variables: pre- and post-operative levels of hemoglobin (Hb); pre- and post-operative levels of sodium; pre- and post-operative levels of potassium; preoperative levels of creatinine.

### Quality assessment

The quality of the included studies was assessed based on the NOS [15], which was recommended by the Cochrane working group on non-randomized research methods [16]. This process includes three major aspects: population selection, comparability of the two groups, and the determination of the outcome. Specifically, each study was assigned a quality score of 0–9, and studies with the NOS score of 7 or more were considered of high quality. Any controversy was again settled by discussion.

### Data analysis

Only those factors reported in more than two articles were entered a meta-analysis software Review Manager Version 5.3 (Nordic Cochrane Centre, Cochrane Collaboration, Copenhagen, Sweden) for analysis. The OR was used to evaluate binary variables, and the MD was used to evaluate continuous variables. ORs or MDs and the corresponding 95% CI were generated to identify a list of potential predictors. Heterogeneity among studies was assessed by the x^2^ tests and *I*^2^ statistics with significance set at *P* <  0.10 or *I*^2^ > 50% [17]. If heterogeneity was found to be significant, random-effects models were used for analysis; otherwise, fixed-effects models were used [18]. The results of the statistically significant factors were graphically summarized using forest plots. When 10 or more studies were provided, a small study publication bias was assessed by the funnel plot analysis. If significant heterogeneity (*I*^2^ > 50%) was detected, it was analyzed further. By sensitivity analysis, anomaly studies were excluded one by one to explore the source of heterogeneity. The value of *P* <  0.05 was considered to be statistically significant. If the factors of interest could not be extracted for meta-analysis or were reported in only one study, the reported clinically significant predictors would be summarized in a systematic review.

## Results

### Search results

Through preliminary search, a total of 219 possible qualified articles were selected. Based on the title and abstract, 15 articles that obviously did not meet the inclusion criteria were excluded first, then 93 duplicates were excluded, and the remaining 111 studies that might meet the inclusion criteria were subsequently reviewed. In the full review, 21 articles that did not use the standard diagnosis of delirium (DSM or CAM) were excluded. In addition, 66 studies that did not have sufficient quantitative data and 9 studies that did not report meaningful results were all excluded. Finally, 15 cohort studies that were identified as meeting the criteria were included in the meta-analysis [19–33] (Fig. 1).

### Study characteristics and quality

A total of 3429 patients were included in this meta-analysis, 368 patients with postoperative delirium and 3061 patients without delirium, with a cumulative incidence of 10.7%. The most used delirium assessment tool was the CAM, which was adopted alone in five studies. Of the fifteen studies included, four were retrospective and eleven were prospective. There was one study on hip arthroplasty, six studies on knee arthroplasty, and the remaining eight studies on hip or knee arthroplasty. The NOS score of the selected studies ranged from 7 to 9, suggesting that the meta-analysis was of high quality (Table S2) (Supplementary Material). Table 1 summarizes the characteristics of each included study.

**Table 1:** The basic characteristics of these 15 included studies and participants

Author (Year)	country	Study types	N	N		Mean Age (years)		Surgery type	Assessment of Delirium	NOS scores
				Delirium	Non-delirium	Delirium	Non-delirium			
**Rogers et al. (1989)** [19]	USA	Prospective	46	13	33	70	69.4	THA, TKA (primary or revision)	DSM-III	7
**Russo et al. (1992)** [20]	USA	Prospective	51	21	30	71.6	65.6	TKA (primary)	DSM-III	8
**Fisher et al. (1995)** [21]	Canada	Prospective	80	14	66	NR	NR	THA, TKA (primary)	CAM	8
**Freter et al. (2005)** [22]	Canada	Prospective	132	18	114	NR	NR	THA, TKA (primary)	CAM	9
**Lowery et al. (2007)** [23]	UK	Prospective	94	14	80	77.2	76.3	THA, TKA (primary)	CAM	7
**Priner et al. (2008)** [24]	France	Prospective	101	15	86	78.2	72.8	THA, TKA (primary)	CAM	7
**Jankowski et al. (2011)** [25]	USA	Prospective	418	42	376	74.76	72.74	THA, TKA (primary)	CAM	7
**Cerejeira et al. (2012)** [26]	UK	Prospective	101	37	64	73.6	72.7	THA (primary)	DSM-IV	7
**Flink et al. (2012)** [27]	USA	Prospective	106	27	79	72.9	73.7	TKA (primary or revision)	DSM-IV, CAM	9
**Chung et al. (2015)** [28]	Korea	Retrospective	365	11	354	NR	NR	TKA (primary)	DSM-IV, CAM	8
**Wang et al. (2016)** [29]	Korea	Retrospective	265	49	216	73.6	69.6	TKA (primary)	DSM-IV, CAM	7
**Huang et al. (2017)** [30]	Singapore	Retrospective	1016	6	1010	78	67	TKA (primary)	DSM-IV	7
**Chen et al. (2017)** [31]	china	Prospective	212	35	177	81.8	72.2	THA, TKA (primary)	DSM-IV, CAM	7
**Peng et al. (2019)** [32]	china	Prospective	272	55	217	74.5	72.1	THA, TKA (primary)	DSM-V	7
**Kijima et al. (2020)** [33]	Japan	Retrospective	170	11	159	79.5	73	TKA (primary)	CAM, DSM-V	7

*DSM* diagnostic and statistical manual of mental disorders, *CAM* confusion assessment method, *UK* United Kingdom, *USA* United States of America, *THA* Total hip arthroplasty, *TKA* Total knee arthroplasty, *NR* Not Reported, *NOS* Newcastle Ottawa Scale

### Predictors assessed

**Fig. 2:**
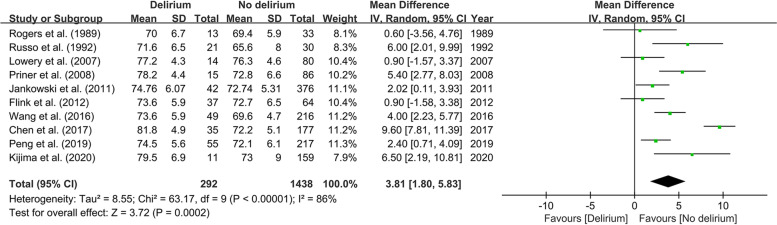
Forest plot of advanced age between Delirium and No Delirium groups

**Fig. 3:**
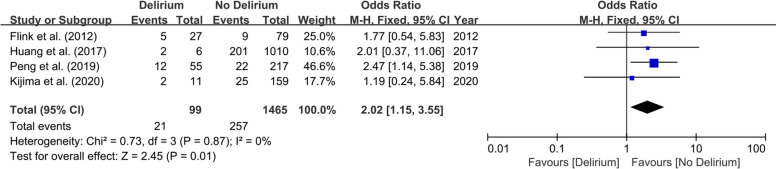
Forest plot of diabetes between Delirium and No Delirium groups

**Fig. 4:**
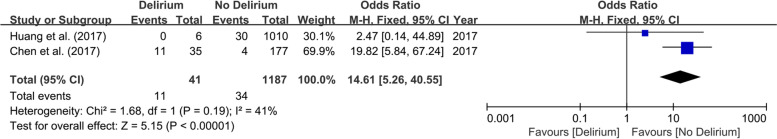
Forest plot of stroke between Delirium and No Delirium groups

**Fig. 5:**
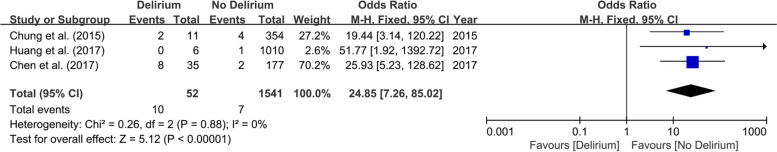
Forest plot of dementia between Delirium and No Delirium groups

**Fig. 6:**
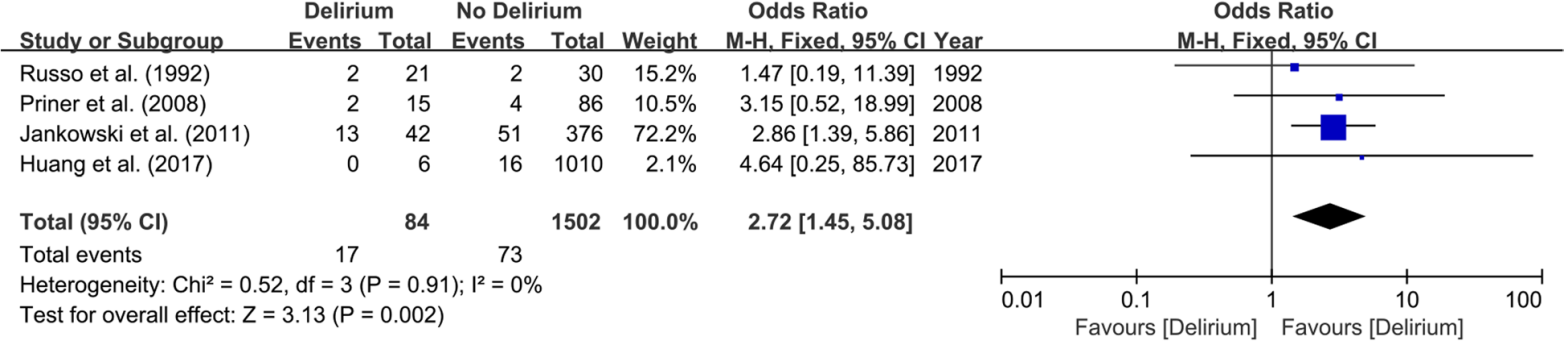
Forest plot of psychiatric illness between Delirium and No Delirium groups

**Fig. 7:**
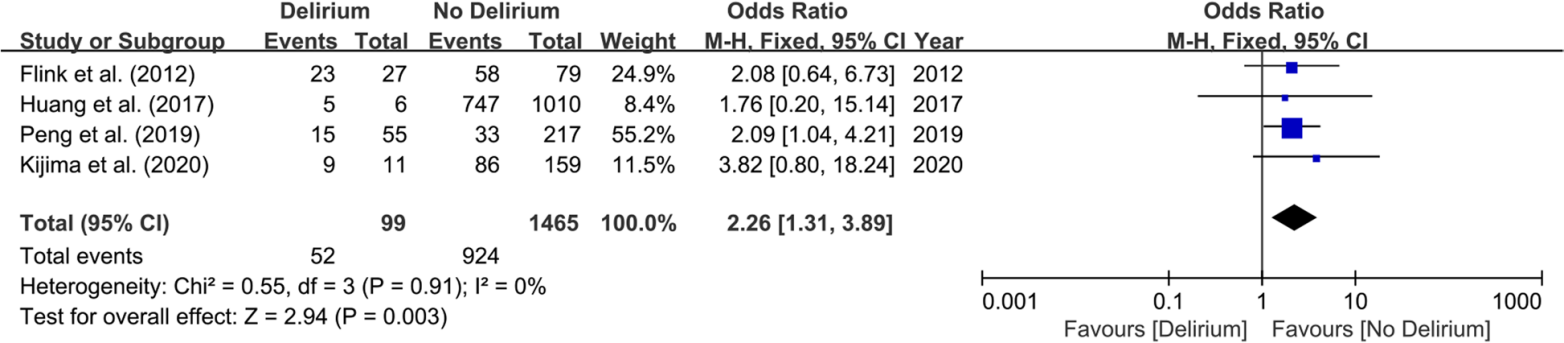
Forest plot of hypertension between Delirium and No Delirium groups

**Fig. 8:**
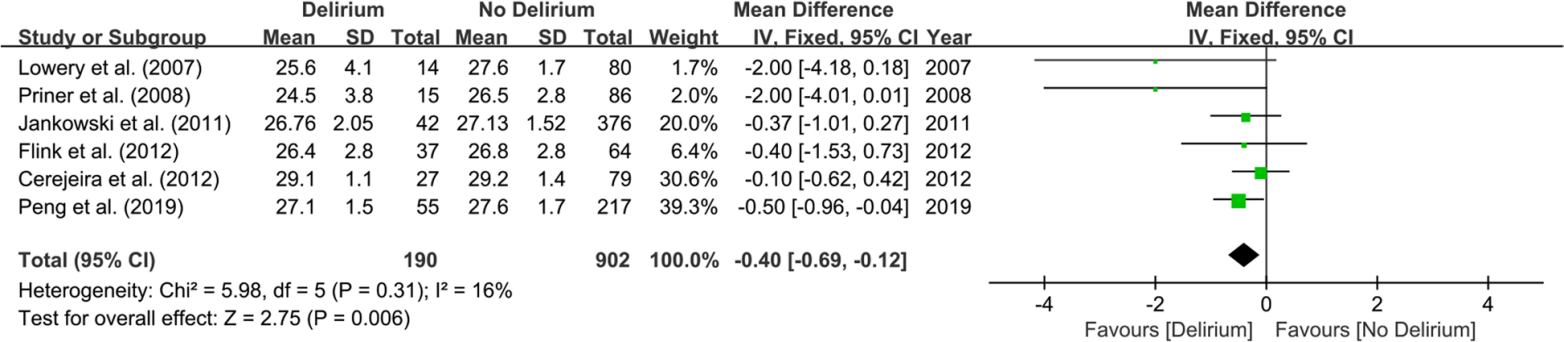
Forest plot of preoperative MMSE scores between Delirium and No Delirium groups

**Fig. 9:**
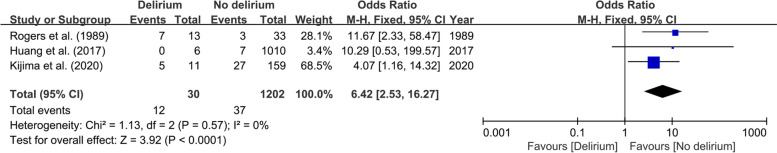
Forest plot of use of sedative-hypnotics between Delirium and No Delirium groups

**Fig. 10:**
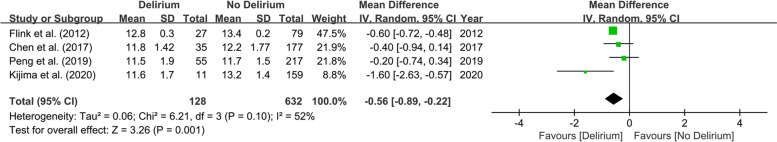
Forest plot of preoperative levels of hemoglobin between Delirium and No Delirium groups

Each potential predictor (*N* ≥ 2) was reported as the effect size of 95% CI in our meta-analyses, and statistically significant factors were shown as funnel plots respectively. All identified predictors were classified into five categories: demographic predictors (Fig. 2), physical status-related predictors (Figs. 3, 4, 5, 6, 7 and 8), surgery-related predictors, drug-related predictors (Fig. 9), and laboratory predictors (Fig. 10). The detailed results for each predictor are shown in Table 2.

**Table 2:** Detailed data on potential predictors for delirium following total joint arthroplasty and the outcomes of meta-analysis

Variable	No. of studies	Pooled OR (95% CI)	Statistical		***P***-value	Effects model
			Heterogeneity			
			***I***^**2**^ (%)	Chi^**2**^		
***Demographic predictors***						
Gender (male)	13	1.14 (0.88, 1.47)	44	0.05	0.33	Fixed
**Advanced age**	10	3.81(1.80, 5.83) ^a^	86	< 0.00001	**0.0002**	Random
Education	2	−0.18 (−1.03, 0.67) ^a^	0	0.68	0.68	Fixed
BMI	4	−0.19 (− 0.37, 0.74) ^a^	0	0.88	0.51	Fixed
Smoking	3	1.18 (0.68, 2.04)	0	0.94	0.56	Fixed
Alcohol abuse	4	1.29 (0.42, 3.91)	83	0.0006	0.65	Random
***Physical status-related predictors***						
**Preoperative MMSE score**	6	−0.40(−0.69, −0.12) ^a^	16	0.31	**0.006**	Fixed
**Diabetes**	4	2.02 (1.15, 3.55)	0	0.87	**0.01**	Fixed
**Hypertension**	4	2.26 (1.31, 3.89)	0	0.91	**0.003**	Fixed
Ischemic heart disease heart disease	2	0.77 (0.17, 3.41)	0	0.73	0.73	Fixed
**Stroke**	2	14.61(5.26, 40.55)	41	0.19	**< 0.00001**	Fixed
**Dementia**	3	24.85 (7.26, 85.02)	0	0.88	**< 0.00001**	Fixed
**Psychiatric illness**	4	2.72 (1.45, 5.08)	0	0.91	**0.002**	Fixed
Hyperlipidemia	2	0.99 (0.55, 1.79)	0	0.97	0.98	Fixed
Surgical site infection	2	5.09 (0.29, 90.26)	81	0.02	0.27	Random
Deep vein thrombosis	2	10.77 (0.04, 2680.35)	92	0.0003	0.4	Random
***Surgery-related predictors***						
General anesthesia	5	1.31 (0.92, 1.86)	48	0.1	0.14	Fixed
Spinal/epidural anesthesia	4	0.69 (0.46, 1.05)	0	0.78	0.08	Fixed
Operation time	3	0.68 (−7.30, 8.66) ^a^	57	0.1	0.87	Random
Intraoperative blood loss	4	8.01 (−71.43, 87.46) ^a^	75	0.008	0.84	Random
Knee arthroplasty	4	1.28 (0.87, 1.88)	6	0.36	0.22	Fixed
Hip arthroplasty	4	0.78 (0.53, 1.15)	6	0.36	0.22	Fixed
***Drug-related predictors***						
**Use of sedative-hypnotics**	3	6.42 (2.53, 16.27)	0	0.57	**< 0.0001**	Fixed
***Laboratory predictors***						
**Preoperative levels of hemoglobin**	4	−0.56 (−0.89, −0.22) ^a^	52	0.1	**0.001**	Random
Preoperative levels of creatinine	2	−0.80 (−7.22, 5.62) ^a^	56	0.13	0.81	Random
Postoperative day 1 sodium	2	−0.17 (−1.73, 1.38) ^a^	0	0.85	0.83	Fixed
Postoperative day 1 potassium	2	0.08 (−0.14, 0.29) ^a^	0	0.52	0.48	Fixed
Postoperative day 1 hemoglobin	2	−0.57 (−1.44, 0.30) ^a^	57	0.13	0.2	Random
Postoperative day 3 sodium	2	−1.27 (−2.67, 0.13) ^a^	0	0.79	0.08	Fixed
Postoperative day 3 potassium	2	−0.06 (−0.23, 0.10) ^a^	0	0.63	0.45	Fixed
Postoperative day 3 hemoglobin	2	−0.66 (−1.88, 0.56) ^a^	61	0.11	0.29	Random

^a^ Mean difference effect estimate, MMSE mini mental state examination, BMI body mass index, CI confidence intervals, I2 inconsistency value, OR odd ratio, P-value probability value, Fixed Fixed-effect model, Random Random-effect model

### Demographic predictors

There were fifteen studies, involving 3429 patients, about demographic characteristics. In the primary analysis, advanced age [19, 20, 23–25, 27, 29, 31–33] (MD 3.81; 95% CI 1.80–5.83; *I*^2^ = 86%; *P* <  0.00001; Fig. 2) was correlated with a high risk of POD. However, gender [19–22, 25–33], BMI [27, 29, 31, 32], smoking [26, 31, 32], alcohol abuse [20, 26, 31, 32], and education [25, 27] showed no significant difference between delirium and no delirium patients.

### Physical status-related predictors

This meta-analysis on physical status-related predictors included eleven studies involving 2906 patients. Patients with diabetes [27, 30, 32, 33] (OR 2.02; 95% CI 1.15–3.55; *I*^2^ = 0%; *P* = 0.87; Fig. 3), stroke [30, 31] (OR 14.61; 95% CI 5.26–40.5; *I*^2^ = 41%; *P* = 0.19; Fig. 4), dementia [28, 30, 31] (OR 24.85; 95% CI 7.26–85.02; *I*^2^ = 0%; *P* = 0.88; Fig. 5), psychiatric illness [20, 24, 25, 30] (OR 2.72; 95% CI 1.45–5.08; *I*^2^ = 0%; *P* = 0.91; Fig. 6), hypertension [27, 30, 32, 33] (OR 2.26; 95% CI 1.31–3.89; *I*^2^ = 0%; P = 0.91; Fig. 7), and lower preoperative MMSE scores [23–27, 32] (MD − 0.40; 95% CI − 0.69−− 0.12; *I*^2^ = 16%; *P* = 0.31; Fig. 8) were significantly related to delirium after TJA. However, patients with ischemic heart disease [30, 33], hyperlipidemia [27, 32], surgical site infection [30, 32], and deep vein thrombosis (DVT) [30, 32] did not show a higher incidence of POD than those without.

### Surgery-related predictors

Ten studies representing 2707 patients were about surgery-related predictors. In this meta-analysis, general anesthesia [25–27, 29, 31], spinal/epidural anesthesia [19, 25, 29, 30], operation time [29, 31, 32], and intraoperative blood loss [29, 31–33] did not show correlation with POD. Notably, we found no significant difference between the incidence of POD after hip arthroplasty and that after knee arthroplasty [19, 24, 25, 32].

### Drug-related predictors

The included studies on drug-related predictors involved 1232 patients. Use of sedative-hypnotics [19, 30, 33] (OR 6.42; 95% CI 2.53–16.27; *I*^2^ = 0%, *P* = 0.57; Fig. 9) indicated a significantly increased risk of POD.

### Laboratory predictors

Six studies representing 1443 patients were involved in laboratory predictors. In the primary analysis, patients with lower preoperative hemoglobin levels [27, 31–33] (MD − 0.56; 95% CI − 0.89−− 0.22; *I*^2^ = 52%; *P* = 0.10; Fig. 10) were more prone to develop delirium following TJA. However, the association of postoperative levels of sodium [31, 33], potassium [31, 33], and hemoglobin [31, 33] with POD in patients undergoing TJA was not significant.

### Publication Bias

**Fig. 11:**
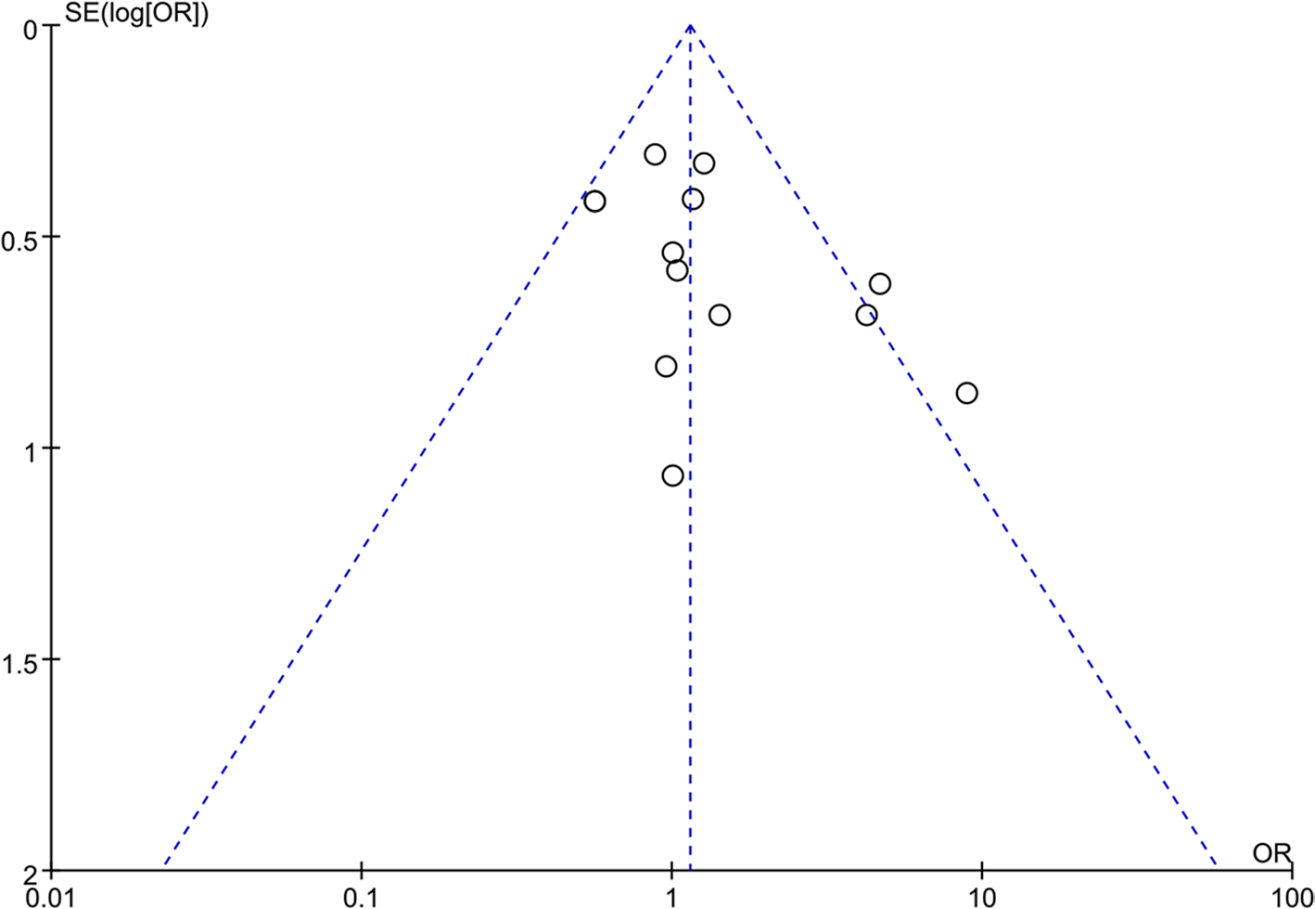
Funnel plot of gender (male) between Delirium and No Delirium groups

The funnel plots for gender were examined visually and its shape was basically symmetrical, demonstrating a low risk of publication bias (Fig. 11).

### Sensitivity analysis

In this meta-analysis, ten factors showed significant heterogeneity (*I*^2^ > 50% or *P* <  0.10); of which five factors (*N* ≥ 3) could be further analyzed. The heterogeneity of four factors changed obviously after the deletion of an article (*I*^2^ reduction > 35%) encompassing alcohol abuse, preoperative hemoglobin levels, operation time, and estimated blood loss. The heterogeneity in estimated blood loss and preoperative hemoglobin levels may be explained by the data recall bias resulting from retrospective studies, and the heterogeneity in operation time and alcohol abuse may be explained by insufficient sample size. However, we cannot definitively explain the high heterogeneity in advanced age, which may be involved in sample size, the adjustment confounders, year of publication, or quality score. In addition, after excluding an article, the statistical significance of two factors changed, covering estimated blood loss and alcohol abuse. Notably, after deleting another article, the *I*^2^ value of advanced age lowered to 47%, and the significance did not change. The results of the sensitivity analysis are presented in Table S3 (Supplementary Material).

### Systematic review

An additional 24 predictors that might be related to POD were summarized in this systematic review (Table S4) (Supplementary Material). These factors were not analyzed in the meta-analysis because they were reported in only one study, comprising clock score ≤ 6, cognitive impairment, substance use, slower simple reaction time (SRT), digit vigilance (DV) reaction time, choice reaction time (CRT), the short form of the Informant Questionnaire on Cognitive Decline in the Elderly (short IQCODE) score > 50, unbalanced inflammatory response, dysfunctional interaction between the cholinergic and immune systems, preexisting obstructive sleep apnea, postoperative day 3 BUN ≥14.9 (mg/dl), hemiplegia, ambulation timing, race, Parkinson, equivalent fentanyl dose, preoperative oxygen partial pressure, preoperative serum total protein level, and preoperative c-reactive protein/albumin ratio (CAR) level.

## Discussion

In this systematic study, we included 15 studies with 31 potential factors. After a preliminary analysis, 9 factors were strongly associated with POD, including comprising advanced age, dementia, hypertension, diabetes, stroke, psychiatric illness, use of sedative-hypnotics, lower preoperative levels of hemoglobin, and lower preoperative mini-mental state examination score. Twelve studies were included in the systematic review, of which 24 factors were additionally correlated with POD using single studies. The above results indicate that close monitoring of the risk factors for POD after TJA is conducive to early prevention, and is of great significance for clinical prognosis, patient stratification and further study of the disease.

POD remains a very common, acute, under-recognized problem in older adults following total joint arthroplasty (TJA) and is associated with a variety of severe cognitive and functional disorders. Given the aging population, the economic and health burden of POD is very likely to increase. Although the underlying pathologic mechanisms between delirium and these adverse outcomes are uncertain, there is no doubt that POD is highly distressing to patients, family members, and providers, indicating an urgent need for prevention of POD through identifying effective predictors [34]. The current meta-analysis extensively analyzed the early predictors of postoperative delirium. Thirty-one predictors were available for meta-analysis, of which 9 predictors were statistically significant: advanced age, dementia, hypertension, diabetes, stroke, psychiatric illness, use of sedative-hypnotics, lower preoperative levels of hemoglobin, and lower preoperative MMSE score.

### Demographic predictors

Advanced age has been recognized as a well-established predictor for POD [35, 36]. Consistently, the results of our meta-analysis showed that patients with POD were 3.8 years older on average than those without. Furthermore, the average age of the population undergoing TJA is 71 years, and most patients are over 65 years old according to a previous study [37]. Therefore, the patient population under this study is at a high risk of POD. The high incidence of POD in elderly patients may be attributed to higher comorbidities, age-related changes in organ and brain composition, pharmacodynamics, renal function, and metabolism [38]. However, Valerio et al. [39] found that comorbidities, rather than age itself, are responsible for the increase in POD. This suggests that the surgeon should work closely with the geriatrician to comprehensively assess the patient’s preoperative risk, and aging should not be a limitation of joint replacement.

Among other demographic predictors, gender, BMI, education, and smoking were not found to be significantly related to the occurrence of POD. Contrary to our hypothesis and previous literature [31, 32, 40], this analysis did not verify a significant correlation between alcohol abuse and POD, but there was significant heterogeneity. Through sensitive analysis, we found one study [26] with a small sample size had a great influence on the pooled result. After the exclusion of this study, the *I*^2^ value lowered to 0%, and the significance changed, indicating alcohol abuse was still likely to be a potential predictor of POD.

### Physical status-related predictors

This meta-analysis indicated that diabetes, stroke, and hypertension were risk factors for POD. The mechanism linking diabetes and delirium might include an increase in proinflammatory cytokines caused by hyperglycemia or subacute or chronic oxidative stress [41]. Diabetes has been shown to be an inflammatory process associated with elevated levels of interleukin-1, interleukin-6, C-reactive protein, and tumor necrosis factor-α throughout the body [42–44]. Notably, a growing body of literature supports the role of inflammatory cytokines in the development of delirium [45, 46]. As for stroke, the relationship between a history of stroke and delirium is well established [47, 48]. Guo et al. [49] found that a history of stroke was an independent risk factor for POD in patients undergoing total hip arthroplasty. Consistent with our findings, Muaaz et al. [50] found that hypertension is a risk factor for POD. The present study showed that multiple comorbidities significantly increased the risk of POD after TJA. This fact suggested that the POD group tended to have more comorbidities and poorer preoperative physical conditions than the control group. Previous research supported the idea that preoperative health status was a major consideration for postoperative adverse events [51].

We also found that preoperative cognitive impairment assessed by MMSE could predict the occurrence of POD. MMSE, as a preliminary screening, can detect people who are less likely to be delirious with about 93–97% accuracy [52]. In this meta-analysis, the mean MMSE score in the case group was only 0.4 points lower than that in the control group, emphasizing that even mild cognitive impairment increased the risk for delirium. Our meta-analysis found a significant correlation between psychiatric illness and POD, which further suggests that patients’ preoperative mental state is an important predictor of POD. Therefore, cognitive testing should be part of a standardized procedure for the preoperative clinical assessment of TJA patients. It was also believed that the pathophysiological mechanisms of delirium may be like some neurodegenerative processes (such as dementia), both of which involved abnormal inflammatory responses or dysfunction of the cholinergic system [26, 53]. The onset of delirium reflects the underlying vulnerability of the brain, cognitive impairment, and an increased risk of dementia in the future [54]; Similarly, dementia is also an important predictor of POD, as demonstrated by this meta-analysis. In the meantime, we must point out that MMSE, as a relatively insensitive clinical cognitive measurement tool, could not be capable of detecting some subtle cognitive changes. However, these slight cognitive changes may still indicate a high risk for POD [27], suggesting that we must use more sensitive tools to detect these subtle cognitive changes. Otherwise, this may lead to irreversible consequences as patients age.

### Surgery-related predictors

In our analysis, under normal surgical conditions, surgery-related factors, such as blood loss, type of anesthesia and operation time, did not seem to have a significant effect on the occurrence of POD after TJA. It was worth noting that there was a high degree of heterogeneity in studies on intraoperative blood loss and operation time, which indicated that the results should be treated with caution. According to the previous research and this fact, we believed that the factors related to surgery still should not be ignored, but it also suggested that the incidence of POD could be reduced by standardizing surgical operation and improving surgical management [10, 55].

Although hip surgery tended to resulted in greater perioperative blood loss and longer time without movement, the results of our meta-analysis showed no significant difference in delirium rates between hip and knee replacement patients. This result is consistent with a systematic review of joint replacement by J.E. et al. [5].

### Drug-related predictors

Our results confirmed that the use of sedative-hypnotics could contribute to POD after TJA. In recent years, benzodiazepine receptor agonists (benzodiazepines and non-benzodiazepines) have been reported to cause cognitive decline and delirium, which supported our findings [55, 56]. Excitingly, new insomnia drugs have been found that might reduce the risk of delirium, such as ramelteon and suvorexant, which selectively target receptors in the pineal gland and hypothalamus (melatonin and orexin receptors), respectively. Furthermore, the potential value of ramelteon and suvorexant against delirium was further verified in a randomized clinical trial [57, 58]. Therefore, for patients with a high risk of POD, it may be considered to replace benzodiazepine receptor agonists with these drugs preoperatively.

It is reported that 12–39% of the occurrence of POD in the elderly is related to medications. Due to pharmacodynamics and pharmacokinetics changes, the high prevalence of multiple drugs, and the existence of coexisting diseases, drug-induced delirium is becoming more common in this population [59]. Strengthening patient consultation, improving medication management, and reducing multi-drug use may be beneficial to the alleviation of this problem.

### Laboratory predictors

Our meta-analysis found a significant correlation between low preoperative hemoglobin levels and POD. Recent studies have shown that anemia was significantly associated with cognitive decline and the development of dementia [60, 61]. Furthermore, studies have suggested that anemia is an independent risk factor for delirium in hospitalized elderly patients [62]. A possible explanation is that low hemoglobin levels reflect the inflammatory state associated with chronic disease, and inflammation may play an important role in the pathological mechanism of delirium [63, 64]. Therefore, low preoperative hemoglobin levels can indirectly predict the high incidence of POD. Notably, there is still no conclusive evidence that correcting preoperative hemoglobin levels can reduce the risk of postoperative delirium [33]. Therefore, for elderly patients with low preoperative hemoglobin levels, it is necessary to reduce other potential risk factors of POD and take preventive measures in advance.

Regarding electrolyte disturbances, there was still no consensus on the role of different electrolyte disturbances in POD. Our meta-analysis found no significant difference in sodium and potassium levels between POD patients and non-POD patients on day 1 and 3 postoperatively. However, Shiiba et al. [65] pointed out that disturbances in sodium levels could be a predictor of POD. Given that potassium and sodium levels are associated with dehydration and anemia, further analysis is needed to assess their true association with delirium.

### Limitations

Some limitations should be emphasized. First, criteria for diagnosing delirium were not uniform across studies and have varied over time, which could lead to bias. Although publication bias and sensitivity analyses have been performed to consolidate the reliability of the results, these data still required careful interpretation. Second, this meta-analysis was limited in accurately determining the relative magnitude of the predictive power of different factors, as this would inevitably be influenced by changes in important methodological factors (such as length of follow-up and experience of the assessors) in different studies. Third, there were no randomized controlled trials in the included literature, which might affect the quality of the results. Fourth, because sufficient separate data on POD in primary total hip arthroplasty or revision arthroplasty were not available, we could not perform relevant subgroup analyses, although they were important indicators that should be evaluated. Considering the above limitations, more well-designed studies which focus the predictors of the POD following TJA are required in the future.

## Conclusions

In summary, this systematic review and meta-analysis, after thorough induction and extensive analysis, provided many valuable predictors of POD after TJA. Several established predictors may be promising not only to predict the risk of developing POD in elderly patients with TJA, but also to refine the clinical treatment process. Our findings suggested that advanced age, dementia, hypertension, diabetes, stroke, psychiatric illness, use of sedative-hypnotics, lower preoperative levels of hemoglobin, and lower preoperative MMES score may contribute to the early prediction of POD after TJA. Further large-sample, high-quality, and well-documented prospective studies are necessary to support these findings.

## Supplementary Information

**Additional file 1: Supplementary Table 1.** Search strategy for PubMed.

**Additional file 2: Supplementary Table 2.** Methodological quality assessment of included studies by Newcastle–Ottawa scales.

**Additional file 3: Supplementary Table 3.** Results of sensitive analysis for variables.

**Additional file 4: Supplementary Table 4.** Reported predictors for postoperative delirium in patients undergoing total joint arthroplasty.

## Data Availability

Not applicable.

## Abbreviations

POD: Postoperative delirium
TJA: Total joint arthroplasty
OR: Odds ratio
MD: Mean difference
NOS: Newcastle-Ottawa Scale
CI: Confidence interval
PRISMA: Preferred Reporting Items for Systematic reviews and Meta-Analyses
DSM: Diagnostic and Statistical Manual of Mental Disorders
CAM: Confusion Assessment Method
BMI: Body mass index
MMSE: Mini-Mental State Evaluation
DVT: Deep vein thrombosis
SRT: Simple reaction time
DV: Digit vigilance
CRT: Choice reaction time
IQCODE: Informant Questionnaire on Cognitive Decline
CAR: C-reactive albumin ratio
