# Supplementary material for: Predictors of postoperative delirium in elderly patients following total hip and knee arthroplasty: a systematic review and meta-analysis
Source: BMC Musculoskelet Disord. 2021 Nov 12;22:945. doi: 10.1186/s12891-021-04825-1 (PMC8588632; doi:10.1186/s12891-021-04825-1)
Supplement: Supplementary file 1 — Additional file 1: Supplementary Table 1. Search strategy for PubMed. [file 12891_2021_4825_MOESM1_ESM.docx]

| **Database** | **Search strategy** |
| --- | --- |
| **PubMed** | ("deliri*"[Title/Abstract] OR "confus*"[Title/Abstract] OR "POCD"[Title/Abstract] OR (("postoperative period"[MeSH Terms] OR ("postoperative"[All Fields] AND "period"[All Fields]) OR "postoperative period"[All Fields] OR ("post"[All Fields] AND "operative"[All Fields]) OR "post-operative"[All Fields]) AND "cognitive disorder"[Title/Abstract]) OR "acute confusional state"[Title/Abstract]) AND ("risk"[MeSH Terms] OR "risk"[All Fields] OR ("predictor"[All Fields] OR "predictors"[All Fields]) OR ("factor"[All Fields] OR "factor s"[All Fields] OR "factors"[All Fields])) AND ("knee"[MeSH Terms] OR "knee joint"[MeSH Terms] OR "hip"[MeSH Terms] OR "joints"[MeSH Terms]) |
|  |  |
|  |  |
|  |  |
|  |  |
|  |  |
