# Supplementary material for: Predictors of postoperative delirium in elderly patients following total hip and knee arthroplasty: a systematic review and meta-analysis
Source: BMC Musculoskelet Disord. 2021 Nov 12;22:945. doi: 10.1186/s12891-021-04825-1 (PMC8588632; doi:10.1186/s12891-021-04825-1)
Supplement: Supplementary file 2 — Additional file 2: Supplementary Table 2. Methodological quality assessment of included studies by Newcastle–Ottawa scales. [file 12891_2021_4825_MOESM2_ESM.docx]

| **Study** | **Selection** | | | | **Comparability** | **Outcome** | | | |
| --- | --- | --- | --- | --- | --- | --- | --- | --- | --- |
|  | **Exposed Cohort** | **Nonexposed Cohort** | **Ascertainment of exposure** | **Outcome of interest** |  | **Assessment of outcome** | **Length of follow-up** | **Adequacy of follow-up** | **Total score** |
| **Rogers et al. (1989)** | ★ | ★ | ★ | ★ | ★ | ★ | − | ★ | 7 |
| **Russo et al. (1992)** | ★ | ★ | ★ | ★ | ★ | ★ | ★ | ★ | 8 |
| **Fisher et al. (1995)** | ★ | ★ | ★ | ★ | ★ | ★ | ★ | ★ | 8 |
| **Freter et al. (2005)** | ★ | ★ | ★ | ★ | ★★ | ★ | ★ | ★ | 9 |
| **Lowery et al. (2007)** | ★ | ★ | ★ | ★ | ★ | ★ | − | ★ | 7 |
| **Priner et al. (2008)** | ★ | ★ | ★ | ★ | ★ | ★ | − | ★ | 7 |
| **Jankowski et al. (2011)** | ★ | ★ | ★ | ★ | ★ | ★ | − | ★ | 7 |
| **Cerejeira et al. (2012)** | ★ | ★ | ★ | ★ | ★ | ★ | − | ★ | 7 |
| **Flink et al. (2012)** | ★ | ★ | ★ | ★ | ★★ | ★ | ★ | ★ | 9 |
| **Chung et al. (2015)** | ★ | ★ | ★ | ★ | ★ | ★ | ★ | ★ | 8 |
| **Wang et al. (2017)** | − | ★ | ★ | ★ | ★ | ★ | ★ | ★ | 7 |
| **Huang et al. (2017)** | − | ★ | ★ | ★ | ★ | ★ | ★ | ★ | 7 |
| **Chen et al. (2017)** | ★ | ★ | ★ | ★ | ★ | ★ | − | ★ | 7 |
| **Peng et al. (2019)** | ★ | ★ | ★ | ★ | ★★ | ★ | ★ | ★ | 9 |
| **Kijima et al. (2020)** | − | ★ | ★ | ★ | ★ | ★ | ★ | ★ | 7 |
