# Supplementary material for: Predictors of postoperative delirium in elderly patients following total hip and knee arthroplasty: a systematic review and meta-analysis
Source: BMC Musculoskelet Disord. 2021 Nov 12;22:945. doi: 10.1186/s12891-021-04825-1 (PMC8588632; doi:10.1186/s12891-021-04825-1)
Supplement: Supplementary file 3 — Additional file 3: Supplementary Table 3. Results of sensitive analysis for variables. [file 12891_2021_4825_MOESM3_ESM.docx]

| **Variables** | **OR or MD and corresponding 95%CI (original)** | **P for heterogeneity** | ***I*^2^** | **The outlier study excluded** | **OR or MD and corresponding 95%CI (afterwards)** | **P for heterogeneity** | ***I*^2^** |
| --- | --- | --- | --- | --- | --- | --- | --- |
|  |  |  |  |  |  |  |  |
|  |  |  |  |  |  |  |  |
| **Advanced age** | 3.81 (1.80, 5.83) | < 0.00001 | 86 | Chen et al. (2017) Priner et al. (2008) | 2.62 (1.39, 3.85) | 0.09 | 47 |
| **Preoperative levels of hemoglobin** | -0.56 (-0.89, -0.22) | 0.1 | 52 | Kijima et al. (2020) | -0.53 (-0.72, -0.34) | 0.3 | 17 |
| **Estimated blood loss** | 8.01 (-71.43, 87.46) | 0.008 | 75 | Kijima et al. (2020) | 43.76 (11.30, 76.23) | 0.81 | 0 |
| **Operation time** | 0.68 (-7.30, 8.66) | 0.1 | 57 | Peng et al. (2019) | -2.16 (-6.82, 2.50) | 0.5 | 0 |
| **Alcohol abuse** | 1.29 (0.42, 3.91) | 0.0006 | 83 | Cerejeira et al. (2012) | 2.09 (1.23, 3.55) | 0.37 | 0 |
| *MD* mean difference effect estimate, *OR* odd ratio, *CI* confidence intervals, *I^2^* inconsistency value, *P-Value* probability value | | | | | | | |
