# Supplementary material for: Predictors of postoperative delirium in elderly patients following total hip and knee arthroplasty: a systematic review and meta-analysis
Source: BMC Musculoskelet Disord. 2021 Nov 12;22:945. doi: 10.1186/s12891-021-04825-1 (PMC8588632; doi:10.1186/s12891-021-04825-1)
Supplement: Supplementary file 4 — Additional file 4: Supplementary Table 4. Reported predictors for postoperative delirium in patients undergoing total joint arthroplasty. [file 12891_2021_4825_MOESM4_ESM.docx]

| **Study** | **Predictors for postoperative delirium** |
| --- | --- |
| **Fisher et al. (1995)** | Clock Score≤6 |
| **Freter et al. (2005)** | Cognitive impairment, substance use |
| **Lowery et al. (2007)** | Slower simple reaction time (SRT), digit vigilance (DV) reaction time, choice reaction time (CRT) |
| **Priner et al. (2008)** | The short form of the Informant Questionnaire on Cognitive Decline in the Elderly (short IQCODE) score>50 |
| **Jankowski et al. (2011)** | Activities of daily living (ADL) scores, instrumental activities of daily living (IADL) scores, auditory verbal learning test (AVLT) scores, stroop color-word test scores, controlled word association test (COWAT) scores |
| **Cerejeira et al. (2012)** | Unbalanced inflammatory response, dysfunctional interaction between the cholinergic and immune systems |
| **Flink et al. (2012)** | Preexisting obstructive sleep apnea |
| **Chung et al. (2015)** | Postoperative day 3 BUN≥14.9 (mg/dl) |
| **Wang et al. (2017)** | Hemiplegia, ambulation timing |
| **Huang et al. (2017)** | Race, parkinson |
| **Chen et al. (2017)** | Equivalent fentanyl dose, preoperative oxygen partial pressure, preoperative serum total protein level |
| **Peng et al. (2019)** | Preoperative C-reactive protein/albumin ratio (CAR) level |
